# Effects of stem cell transplantation on cognitive decline in animal models of Alzheimer’s disease: A systematic review and meta-analysis

**DOI:** 10.1038/srep12134

**Published:** 2015-07-10

**Authors:** Zhe Wang, Weijun Peng, Chunhu Zhang, Chenxia Sheng, Wei Huang, Yang Wang, Rong Fan

**Affiliations:** 1 Department of Integrated Chinese and Western Medicine, The Second Xiangya Hospital, Central South University, Changsha 410011, P.R. China; 2 Institute of Integrated Medicine, Xiangya Hospital, Central South University, Changsha 410008, P.R. China

## Abstract

Alzheimer’s disease (AD), an irreversible progressive neurodegenerative disease, causes characteristic cognitive impairment, and no curative treatments are currently available. Stem cell transplantation offers a powerful tool for the treatment of AD. We conducted a systematic review and meta-analysis of data from controlled studies to study the impact of stem cell biology and experimental design on learning and memory function following stem cell transplantation in animal models of AD. A total of 58 eligible controlled studies were included by searching PubMed, EMBASE, and Web of Science up to April 13, 2015. Meta-analysis showed that stem cell transplantation could promote both learning and memory recovery. Stratified meta-analysis was used to explore the influence of the potential factors on the estimated effect size, and meta-regression analyses were undertaken to explore the sources of heterogeneity for learning and memory function. Publication bias was assessed using funnel plots and Egger’s test. The present review reinforces the evidence supporting stem cell transplantation in experimental AD. However, it highlights areas that require well-designed and well-reported animal studies.

Alzheimer’s disease (AD), the leading cause of dementia, is a neurodegenerative disease characterized by a loss of neurons in the cerebral cortex and the hippocampus associated with the accumulation of amyloid-β plaques and neurofibrillary tangles, synaptic and neuronal loss, and cognitive decline^1^,^2^. In the United States, an estimated 5.2 million people have AD, and the number of people living with AD is expected to be 13.8 million by 2050^3^. Worldwide, more than 35 million people have been suggested to suffer from AD today, with predictions that there could be more 125 million patients with AD by 2050^4^. The increasing prevalence of Alzheimer’s disease represents a global challenge at personal, social, and economic levels^5^.Unfortunately, no effective therapeutic strategies are available today for AD to slow or stop the malfunction and death of neurons in the brain that cause AD symptoms and eventually make the disease fatal^3^,^6^,^7^.

Recently, with the advent of stem cell technologies and the ability to transform these cells into different types of central nervous system neurons and glial cells, the transplantation of stem cells, including embryonic stem cells (ESCs), induced pluripotent stem cells (iPSCs), and tissue-derived stem cells such as bone marrow (BM)-and adipose-derived stem cells, has received considerable attention as a potential approach to treat various diseases, including AD^8^. There is now considerable preclinical literature on the possible benefits of stem cell transplantation against AD. Stem cell transplantation could lead to improvement in cognitive and memory performances and increased neuronal survival as a result of decreases in β-amyloid plaques, neurofibrillary tangles, neurodegeneration, and microglia activation in animal models of AD^9^,^10^,^11^,^12^. Moreover, neural stem cells could ameliorate the complex behavioral deficits associated with widespread AD pathology via BDNF^13^. *In vitro* and *in vivo*, the transplantation of bone marrow-derived mesenchymal stem cells (BM-MSCs) has been shown to ameliorate Aβ-induced neurotoxicity and cognitive decline by inhibiting apoptotic cell death and oxidative stress in the hippocampus^12^. It has also been demonstrated that human umbilical cord blood-derived mesenchymal stem cell (hUCB-MSCs) transplantation could rescue the impaired memory function in AD mice by reducing apoptosis and modulating oxidative stress^14^. Human amniotic epithelial cell (HAEC) transplantation could significantly ameliorate spatial memory deficits in transgenic mice as well as the increased acetylcholine levels and amount of hippocampal cholinergic neuritis^15^.

Although stem cells represent a strong candidate for treating AD in animal models, further studies are needed to determine the appropriate conditions to improve the therapeutic effects on AD, such as the ideal type of stem cell, the best source from which to harvest those stem cells for implantation, the number of cells needed, and the site of transplantation of the implanted cells. Moreover, to inform decisions regarding the design and execution of subsequent clinical trials, whether the magnitude of integrative and protective effects is large enough to be potentially clinically meaningful and whether reports of efficacy in animal models are potentially biased in favor of positive results also need to be investigated.

Therefore, we conducted a systematic review and meta-analysis of data from controlled studies to test the efficacy of stem cells as a treatment in animal models of AD. The aims of the current study were to (1) identify all animal experiments describing the efficacy of stem cell-based therapies in models of AD, (2) systematically review the literature describing the effect of stem cell-based therapies on cognitive impairment in animal models of AD, (3) perform a meta-analysis using the DerSimonian and Laird random effects model, (4) provide empirical evidence of the biological factors associated with greater efficacy, and (5) provide an assessment of the presence and impact of possible publication bias.

## Methods

### Search strategy

Using pre-specified inclusion and exclusion criteria (see below), we identified all publications reporting the efficacy of stem cells in an *in vivo* animal model of AD by searching three electronic databases (PubMed, EMBASE, and Web of Science; April 13, 2015) using the search strategy “(stem cell OR stem OR hematopoietic OR mesenchymal) AND (Alzheimer’s disease OR dementia OR senile dementia),” with various Boolean operators.

Searches of the databases using the search strategy were performed independently by two individuals. The bibliographies of relevant articles were used to identify further relevant publications. Abstracts were independently screened by two reviewers to identify those that met our inclusion criteria (see below), with differences resolved by discussion with a third reviewer.

### Inclusion and Exclusion Criteria

We included studies in which learning and memory functional outcome in a group of animals with AD and treated with allogeneic or autologous stem cells was compared with learning and memory functional outcome in a control group of animals treated with placebo (saline, culture medium or similar vehicle). Labeling or transfection with markers for cellular tracing and imaging (e.g., green fluorescent protein, lacZ, bromodeoxyuridine, superparamagnetic iron oxide particles) was included.

We excluded studies using a co-treatment with another therapy or cell type. Studies that used concomitant injection with other cell types or adjuvant neuroprotective agents were also excluded.

To be included in the meta-analysis, outcomes must have been reported as a behavioral outcome, and the number of animals in each group, the mean effect size and variance must have been reported. Disagreements between investigators were resolved by consensus after discussion.

### Data extraction

The following items were extracted by two investigators from each included study: reference details (publication year, and name); recipient animal (rat strain, sex); AD model; stem cells (donor species and tissue source); intervention regime (administration route and number of injections); and cognitive outcome assessments.

We extracted details of the individual study characteristics from each publication, and when a single publication reported more than 1 experiment, these data were extracted and treated as independent experiments. When neurobehavioral tests were performed serially, we only extracted data for the final time point.

In cases of missing data, we contacted the authors and requested the additional information. If data were expressed only graphically, numerical values were requested from the authors; if a response was not received, digital ruler software was used to estimate numerical values from the graphs. If the required data were not presented or obtainable, the study was excluded from the analysis.

### Methodological quality of studies

We assessed the methodological quality of the included studies using the criteria based on a checklist as previously described with minor modifications^16^. The checklist was comprised of the following 17 items, (1) Was the research question specified and clear? (2) Were the outcome measures relevant for AD research? (3) Are the characteristics (species, background/generation, sex [and distribution], and age) of the study population clear? (4) Was the correct control group present? (5) Were the groups similar at baseline (if not randomized, were they similar with respect to characteristics such as weight and sex)? (6) Was the experiment randomized? (7) Was the type of stem cell(s) mentioned? (8) What was the age at which stem cell transplantation was started? (9) Was the duration of stem cell transplantation clear and specified? (10) Was the number of stem cells mentioned? (11) Was the administration route specified? (12) Were the methods used for outcome assessment the same in both groups? (13) Were the dropouts described for each group separately? (14) Was the outcome assessment blinded? (15) Was the outcome assessment randomized across the groups? (16) Was the total number of animals included in the statistical analyses clear? (17) Was the age at which the animals were sacrificed mentioned?

The quality of all studies was assessed independently by two reviewers. It should be noted that the quality assessment mainly assessed the quality of the reporting. Negative judgment did not necessarily indicate that the experiment was performed insufficiently; it indicated that there was inadequate information to assess the quality. (Table 3)

### Statistical analysis

According to the *Cochrane Handbook for Systematic Reviews of Interventions,* the global estimated effect of statin treatment on cognitive outcome was determined by calculating the standardized mean difference (SMD) and 95% confidence intervals (CI) using a random effects model to avoid heterogeneity^17^. SMD is used as a summary statistic in meta-analyses when studies assess the same outcome but measure the outcome in a variety of ways (e.g., multiple studies measuring depression but using different psychometric scales). Within- and between-study variation or heterogeneity was assessed using Cochran’s *Q*-statistic^18^,^19^, with a significant *Q*-statistic (*P *< 0.10) indicating heterogeneity among studies. Heterogeneity was also assessed using the *I*^2^ statistic. Values of 25, 50 and 75% were considered to represent low, moderate and considerable heterogeneity, respectively^20^. For studies comparing different doses and/or times of drug administration with a single control group, we compared control group data with pooled data from all experimental groups.

Stratified meta-analysis was used to explore the influence of the potential factors on the estimated effect size^21^. Differences in mean effect sizes were assessed, partitioning heterogeneity using the *χ*^*2*^ distribution with n-1 degrees of freedom (df), and the significance level was set at *P* < 0.05. Meta-regression analyses were conducted to reveal potential sources of heterogeneity, as described in a previous study^22^. The meta-regression was univariate rather than multivariate, and we calculated adjusted R^2^ values to explain the proportion of the observed variability in the observed effect size for a group of experiments explained by variation in the independent variable in question^16^.

The presence of small effect sizes was investigated using funnel plots and Egger’s test. For Egger’s test, a *P*-value of *<* 0.10 was considered to indicate the presence of a small effect size^18^.

All statistical analyses were performed using Review Manager (version 5.3, The Nordic Cochrane Centre, The Cochrane Collaboration, Copenhagen, Denmark) and Stata software (version 13.0, StataCorp, College Station, TX, USA).

## Results

### Study Selection

**Figure 1:**
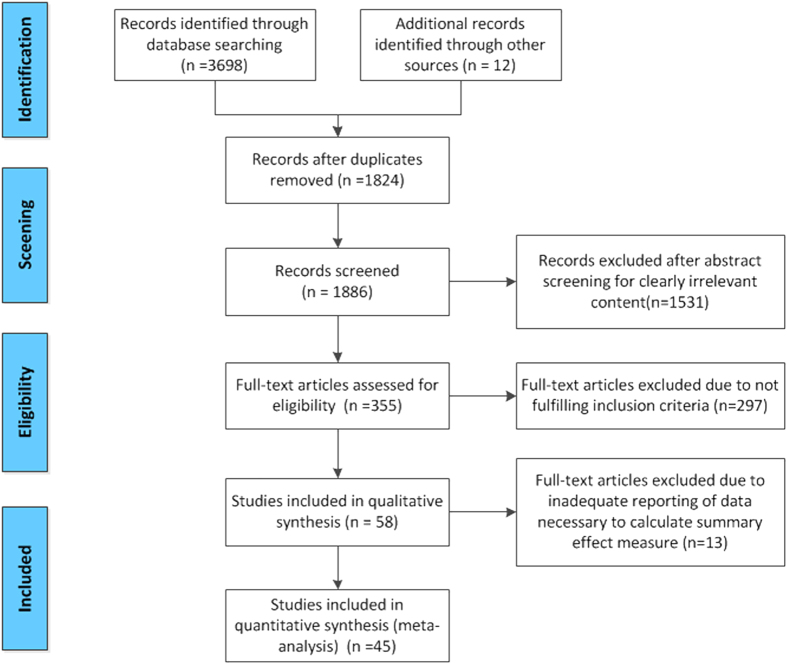
Flow diagram of the search process.

Our review identified 3710 publications; 58 met our pre-specified inclusion criteria. Of these, 12 studies were excluded due to inadequate reporting of the data necessary to calculate the summary effect measure outcome. Therefore, our meta-analysis was based on 46 publications, which included 52 comparisons of learning functions and 73 comparisons of memory functions (Fig. 1).

### Description of Studies

Table S1 shows the characteristics of the included studies. A large variation existed in most study characteristics. Twenty-three studies were performed with transgenic mice, sixteen used Aβ-infused rats, four used age-induced Alzheimer’s model rats, twelve used chemically induced Alzheimer’s model rats, and four used surgery-induced AD models, including fimbria-fornix transaction^23^ and olfactory bulbectomy^24^. Babaei *et al.* employed two methods to establish their AD model^25^. Thirty-eight studies used only males, three studies used only females, five studies used both genders, and twelve studies did not mention which gender was used.

Mesenchymal stem cells (MSCs) (24 studies) derived from human umbilical cord blood, bone marrow, human placenta amniotic membrane, and human placenta were the most frequently investigated, followed by neural stem cells (NSCs, 23 studies). Stereotaxic transplantation (50 studies) was the most frequently reported route of administration. In addition, the water maze and Y-maze tests were the most frequently used measures of cognitive function. The number of cells injected ranged from 2 × 10^6^ to (2–3) × 10^2^.

### Methodological quality of studies

The median quality score was 13 items of 20 (range 10–18). Although randomization, blinding, and description of the number of dropouts are key quality measures in the quality assessment of clinical trials, only 26 included studies randomly allocated the experimental units across the treatment groups. Eight studies described the number of dropouts per group. Moreover, the majority of included studies failed to clearly report the items, such as the age at which stem cell transplantation was started, the duration of stem cell transplantation, and the age at which the animals were sacrificed (Table S2).

### Meta-analysis

**Figure 2:**
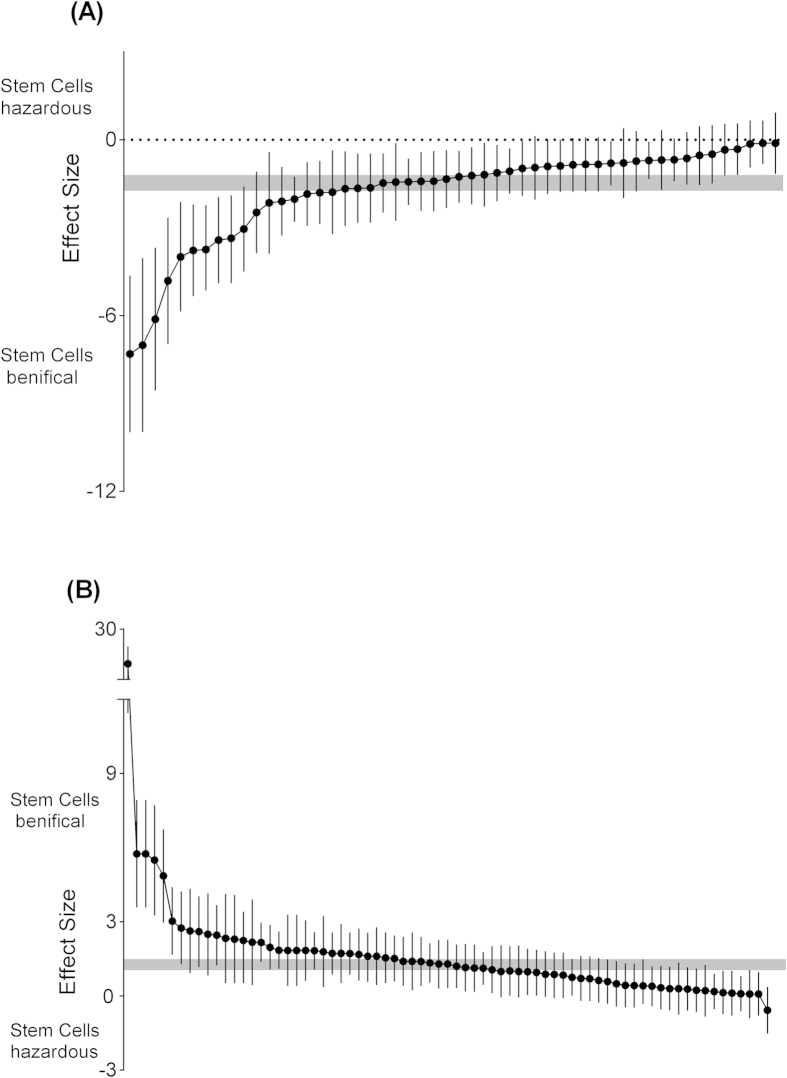
Summary of the data included in the meta-analysis of the use of stem cells to treat AD with individual comparisons ranked according to their effect on learning (**A**) and memory (**B**) function. The shaded gray bar represents the 95% confidence limits of the global estimate. The vertical error bars represent the 95% CIs for the individual estimates.

Fifty-two comparisons of 37 included studies^10^,^12^,^14^,^25^,^26^,^27^,^28^,^29^,^30^,^31^,^32^,^33^,^34^,^35^,^36^,^37^,^38^,^39^,^40^,^41^,^42^,^43^,^44^,^45^,^46^,^47^,^48^,^49^,^50^,^51^,^52^,^53^,^54^,^55^,^56^,^57^,^58^ involving 1045 animals examined the effect of stem cell transplantation on the impaired learning function in animal models for AD using the Morris water maze test, Y-maze and the radial arm maze. The pooled analysis indicated that animals in the treatment group significantly improved in learning recovery more than animals in the control group (SMD = −1.47, 95% CI −1.76 to −1.20, P < 0.0001). There was evidence of moderate heterogeneity among studies (Chi^2^ = 175.5, df = 51 [P < 0.00001]; I^2^ = 71%) (Fig. 2A). Seventy-three comparisons of 36 included studies^10^,^26^,^27^,^29^,^30^,^31^,^32^,^33^,^34^,^36^,^37^,^38^,^39^,^40^,^41^,^43^,^44^,^45^,^46^,^48^,^49^,^50^,^51^,^52^,^53^,^54^,^55^,^58^,^59^,^60^,^61^,^62^,^63^,^64^,^65^,^66^ involving 1495 animals studied the effect of stem cell transplantation on the impaired memory function in animal models for AD using the Morris water maze test, Y-maze, novel object recognition task and single-trial passive avoidance test. The pooled analysis indicated that animals in the treatment group significantly improved in memory recovery relative to animals in the control group (SMD = 1.27, 95% CI 1.04 to 1.49, P < 0.0001). There was evidence of moderate heterogeneity among the studies (χ^2^ = 244.51, df = 72 [P < 0.00001]; I^2^ = 71%) (Fig. 2B).

### Stratified meta-analysis

In the stratified meta-analysis, the impact of study characteristics on the effect sizes was examined. For learning function, the stratified analysis showed that significant differences in effect sizes were observed relative to recipient species (P = 0.02), recipient sex (P = 0.0008), number of cells injected (P = 0.03), and quality scores (P *<* 0.0001). No significant differences in effect sizes were observed relative to the type of AD model (P = 0.1), donor species (P = 0.05), type of stem cells (P = 0.38), type of manipulation of stem cells prior to implantation (P = 0.91), and route of cell delivery (P = 0.30) (Table S3.1).

For memory function, the stratified analysis showed that significant differences in effect sizes were observed relative to the type of AD model (P = 0.01), recipient species (P = 0.007), recipient sex (P = 0.003), donor species (P = 0.004), number of cells injected (P = 0.008), and quality scores (P = 0.004). No significant differences in effect sizes were observed relative to the type of stem cells (P = 0.38), type of manipulation of the stem cells prior to implantation (P = 0.36), and route of cell delivery (P = 0.8) (Table S3.2).

### Meta-regression

To further explore heterogeneity among the studies, meta-regression was conducted to investigate the effect of both continuous and categorical characteristics on learning and memory function.

For learning function, we found that the recipient species (Ad R^2^ = 3.72%) and the recipient sex (Ad R^2^ = 2.66%) accounted for a significant proportion of the between-study heterogeneity in studies (Table S3.1). For memory function, the type of AD model (Ad R^2^ = 28.35%), recipient species (Ad R^2^ = 16.96%), recipient sex (Ad R^2^ = 15.85%), donor species (Ad R^2^ = 26.22%), and number of cells injected (Ad R^2^ = 14.63%) were significant sources of heterogeneity (Table S3.2).

### Publication bias

**Figure 3:**
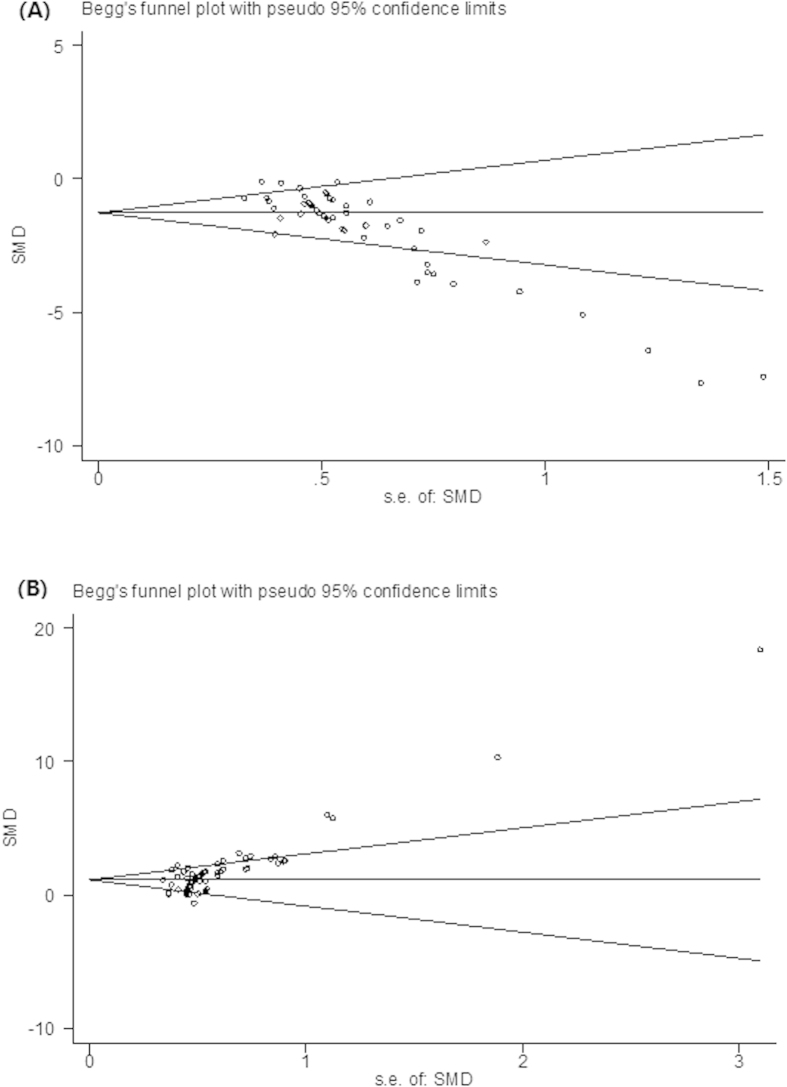
Funnel graph for the assessment of potential publication bias of learning (**A**) and memory (**B**) functions.

Finally, we sought to identify the presence of small study effects, which may have contributed to publication bias. Funnel plots showed asymmetry for both learning and memory function data, indicating the evidence of small study effects (Fig. 3A,B); (Egger regression, <0.0001 and <0.0001, respectively).

## Discussion

Systematic reviews and meta-analyses of animal experiments not only allow for a more objective appraisal of the research evidence than is allowed by the traditional narrative reviews more commonly associated with animal research but also offer a sensible and rational approach to assessing the translational potential of promising experimental interventions before decisions are made to proceed with clinical trials^67^. Therefore, in this study, we intended to provide a detailed systematic review and meta-analysis of the animal literature describing stem cell-based therapy for AD.

Overall, our random-effects meta-analysis results suggested a beneficial effect of stem cell transplantation in experimental AD in terms of promoting learning and memory recovery and reinforced the evidence supporting stem cell-based therapy for experimental AD. For both learning and memory outcomes, there was a broad range of experimental approaches, reflected in the observed moderate heterogeneity. This is typical for systematic reviews in animal studies^67^,^68^ and validates our choice of a random effects model, and our summary estimates should be considered as the average efficacy rather than the best estimate of a single “true” efficacy^16^.

Concerning the study quality, our study quality checklist assesses aspects of both internal and external validity, and we found that the quality of most included studies was poor (13, interquartile range 12–14) and that the global estimated effect may be overestimated because many failed to report blinded and randomized assessments of outcome or describe drop-outs, which are important elements that are generally required in reports of preclinical studies^69^. In addition, we found that higher study quality was associated with lower efficacy for learning and memory functions. This association is consistent with the preclinical data for many other disease therapies, such as stroke and traumatic brain injury, in which higher study quality has repeatedly been associated with lower efficacy^70^,^71^. This association also decreases the confidence in their therapeutic translational potential. However, the overall quality scores accounted for a significant proportion of between-study heterogeneity.

The stratified analysis results showed that studies that did not use transgenic mouse models or the Aβ infusion models were associated with significantly larger effects. AD transgenic mouse models are the most widely used animal models for AD and have yielded significant research breakthroughs and Aβ infusion models have been a useful complement to transgenic approaches to AD neuropathology^72^. We found that the studies that used the fimbria-fornix lesion model showed larger behavioral gains. Of course, these results should be interpreted with caution due to the small sample sizes within the subgroups.

For both learning and memory function, these correlations seem to be the influence of high effect sizes reported in studies in which the sex of the animals used was male and in which neural precursor cells (NPCs) were transplanted into the AD model. In addition, efficacy was higher in the studies in which stem cells were delivered stereotaxically to the lateral ventricle, hippocampus, basal forebrain and cerebral cortex, rather than systematically (including intravenous and intracardiac transplantation). This observation suggests that the local implantation of stem cells into the brain may directly mediate AD pathology in animal models and that the blood-brain barrier (BBB) may mask the benefit provided by stem cells delivered intravenously or intracardially^73^. In addition, studies that used rats as recipient species and donor species led to significantly higher estimates of effect. It should be noted that studies in the subgroup analysis used allogeneic or xenogeneic grafts.

We did not find a dose-response relationship. Subgroup analyses revealed that transplantation of the median number of cells ([1–5] × 10^5^) appeared to have a stronger impact on learning and memory function outcomes. There may have been no dose–response effect, or the doses used in these experiments may have all been large enough to generate maximal responses. When the dose response was formally studied, the authors also found no dose-response effect^52^.

The genetic modification of stem cells prior to transplantation can increase cell survival and make them more effective^74^,^75^, and modified cells could be used for the delivery of factors that can ameliorate neurological disorders^76^. Moreover, studies that used stem cells that over-express different neurotrophic factors, such as BDNF, acetylcholine (Ach), and nerve growth factor (NGF), found that the delivery of these genetically modified stem cells to animal models of AD is safe and effective to restore learning and memory functions, thus suggesting that stem cell-based gene therapy may be a promising treatment for AD^29^,^53^,^57^,^58^,^77^. However, we did not observe that prior gene modification of the implanted cells was associated with larger effects. The underlying mechanism responsible for this should also be investigated in greater depth in the future.

This study has several limitations, which are also observed in previous systematic reviews of animal studies^78^,^79^,^80^. First, the publication bias should be considered. Our search strategy was designed to be exhaustive to identify all potentially relevant published and unpublished studies, and our analysis was only able to include the majority of published studies in this field. The funnel plots and Egger’s test suggested the possibility of publication bias or other small study biases that affected the outcomes^67^. Second, extracting multiple pieces of information from a single publication has the potential to introduce bias into systematic reviews because we have observed the experiments of others and have not conducted experiments of our own; thus, this observational research should be considered to be hypothesis-generating only^16^. Third, we present a series of univariate analyses in which meta-regression might provide more robust insights, but these techniques are not well established; additionally, this analysis might represent an oversimplification, at least for some independent variables^16^. Finally, we limited our analysis to neurobehavioral outcomes, and functional outcome, in combination with histopathology, may be just as important in terms of assessing potential neuroprotective drugs^81^. It is thus worthy of further exploration.

As mentioned in our previous systematic reviews^67^,^82^, to improve the transition from animal experiments to human clinical trials, researchers are strongly recommended to consult and follow the ARRIVE guidelines^83^,^84^ when designing studies and to report full methodological details to allow others to reproduce and validate their results and enable more accurate reviews and meta-analyses. Second, due to the risks of tumorigenesis^85^, further studies are needed to investigate the safety of stem cell transplantation. Third, despite the fact that the gene modification of stem cells prior to transplantation exhibits great potential for the treatment of AD, some major problems remain to be overcome. For example, the more specific and extensive the genetic modification, the longer the stem cells must remain *in vitro*. Finally, the stem cells themselves have therapeutic effects; however, the sustained effects of stem cells require the long-term survival of transplanted cells, and determining the appropriate conditions to improve the therapeutic effects for AD pathology is required^86^.

## Conclusions

The present systematic review and meta-analysis would seem to suggest that stem cells have substantial beneficial effects on learning and memory recovery in animal models of AD. However, without rigorous, robust and detailed pre-clinical evaluation, the results should be interpreted in light of the known limitations in animal experimental design and methodological quality. Therefore, more well-designed and well-reported experimental animal studies are needed.

## Additional Information

**How to cite this article**: Wang, Z. *et al.* Effects of stem cell transplantation on cognitive decline in animal models of Alzheimer's disease: A systematic review and meta-analysis. *Sci. Rep.* **5**, 12134; doi: 10.1038/srep12134 (2015).

## Supplementary Material

Supplementary Information

Supplementary Information

Supplementary Information
