# Supplementary material for: Effects of stem cell transplantation on cognitive decline in animal models of Alzheimer’s disease: A systematic review and meta-analysis
Source: Sci Rep. 2015 Jul 10;5:12134. doi: 10.1038/srep12134 (PMC4498325; doi:10.1038/srep12134)
Supplement: Supplementary Information [file srep12134-s1.pdf]

Effects of stem cell transplantation on cognitive decline in animal models of Alzheimer's disease: A systematic review and meta-analysis

Zhe Wang,MD, Weijun Peng,MD, Chunhu Zhang, PhD, Chenxia Sheng, PhD, Wei Huang, PhD, Yang Wang, PhD , Rong Fan, PhD

Table S1: Study Characteristics of included studies

Authors	AD model	Recipient species	Recipient sex	Donor species	Type of stem cell	Number of cells injected	Route of delivery	Cognitive outcome measure(s)
Wu 2004[1]	OA- infused	rat	male/female	rat	NSCs	$5.4x10^{4}$	Stereotaxic(i.h.)	MWM
Wang 2005[2]	Ibotenic acid-infused NBM lesion	mouse	male	cell line	NSCs (modified)	$(1.6\text{-}8)\ x10^{4}$	Stereotaxic (cortex)	Eight-arm radical maze
Zhao 2005[3]	Unilaterally FF transaction	rat	male	rat	NSCs (modified)	$8\ x10^{5}$	Stereotaxic(latera l ventricle)	MWM
Wang 2006[4]	Ibotenic acid-infused NBM lesion	mouse	male	cell line	NSCs (modified)	$(1.6\text{-}8)\ x10^{4}$	Stereotaxic(i.c.)	Eight-direction maze
Tang 2007[5]	Aβ 1-40 -infused	rat	male/female	cell line	NPCs (modified)	$20\ x10^{6}$	Stereotaxic(i.h.)	MWM
Wu 2007[6]	Aβ 1–40-infused	rat	female	mouse	BMSC	$(2\text{-}3)\ x10^{6}$	Stereotaxic(i.h.)	MWM
Yang 2007[7]	Aβ 1–40-infused	rat	male	rat	NSCs	$5\ x10^{5}$	Stereotaxic(i.h.)	Y-maze
Gu 2008[8]	Left FF transaction	rat	male	rat	NSCs	$1x10^{5}$	Stereotaxic(basal forebrain)	Y-maze

Li&Li 2008[9]	Aβ 1–40 -infused	rat	female	mouse	BMSC (modified)	$(2–3)\times10^5$	Stereotaxic(i.h.)	MWM
Li&Guang2008[10]	1×TgAD	TG mice	male/female	human	hAM-MSCs	$5x10^5$	Systematic(IV)	MWM
Li&Tang 2008[11]	Aβ 1–40 -infused	rat	male	cell line	NSCs (modified)	$5X\ 10^6$	Stereotaxic(i.h.)	MWM
Tang 2008[12]	Aβ 1–40 -infused	rat	male	cell line	NPCs (modified)	$1X\ 10^6$	Stereotaxic(i.h.)	MWM
Wei 2008[13]	Aβ -infused	rat	male	rat	BMSCs (modified)	$8X\ 10^5$	Stereotaxic(latera l ventricle)	MWM
Wu 2008[14]	OA-infused	rat	male	rat	NSCs (modified)	400	Stereotaxic (i.h.&cortex)	MWM
Blurton-Jones 2009[15]	3xTg-AD	TG mice	unclear	mouse	NSCs (modified)	$2X\ 10^6$	Stereotaxic(i.h.)	MWM NORT
Li 2009[16]	natural senile	rat	male	human	BM-MSCs (modified)	$4x10^5$	Stereotaxic(i.h.)	Y-maze
Moghadam 2009[17]	Ibotenic Acid-Induced NBM lesion	rat	male	cell line	NPCs (modified)	$2X\ 10^5$	Stereotaxic(right NBM)	MWM
Xuan 2009[18]	Unilateral FF transection	rat	male	rat	NSCs	$5X\ 10^4$	Stereotaxic(basal forebrain)	Y-maze
He 2010[19]	natural senile	rat	male	Human	BM-MSCs (modified)	$2x10^5$	Stereotaxic(i.h.)	Y- maze
Lee&Lee 2010[20]	Aβ- infused	mouse	unclear	human	hUCB-MSCs	$1x10^4$	Stereotaxic(i.h.)	MWM

Lee&Bae 2010[21]	Aβ1–42- infused	mouse	unclear	mouse	BM-MSCs	$1x10^5$	Stereotaxic(i.h.)	MWM
Lee&Endo 2010[22]	2×TgAD	TG mice	male	mouse	BM-MSCs	$1x10^4$	Stereotaxic(i.h.)	MWM
Li 2010[23]	Aβ1-40- infused	rat	male	cell line	NPCs	$1x10^6$	Stereotaxic(i.h.)	MWM
Pan 2010[24]	192-IgG-saporin-infused	rat	male	rat	NSCs	200-300	Stereotaxic(basal forebrain)	Y-maze
Shen 2011[25]	Aβ1-40- infused	rat	male	rat	NSCs	400	Stereotaxic(latera l ventricular)	MWM
Xue 2011[26]	2×TgAD	TG mice	unclear	human	HAECs	$1.2X 10^5$	Stereotaxic(latera l ventricular)	Six radial water maze
Babaei 2012[27]	natural senile / Ibo-infused NBM Lesion	rat	male	rat	BM-MSCs	$5x10^5$	Stereotaxic(i.h.)	MWM
Esmaeilzade 2012[28]	Aβ1-40- infused	rat	male	rat	EPI-NCSC	$(2\text{-}3)\ x10^5$	Stereotaxic(i.h.)	Y-maze/PAT
Kim 2012[29]	2×TgAD	TG mice	male	human	hASCs	$1x10^6$ $1x10^5$	Systematic(IV) Stereotaxic(i.h.)	MWM
Lee&Lee2012 [30]	2×TgAD	TG mice	male	human	hUCB-MSCs	$1x10^5$	Stereotaxic(i.h.)	MWM

Lee&Lim2012[31]	Ibo-infused	mouse	male	cell line	NSCs (modified)	$2x10^5$	Stereotaxic(i.h.)	MWM
Lee& Schuchman 2012[32]	2×TgAD	TG mice	unclear	mouse	BM-MSCs (modified)	$1x10^7$	Systematic(IV)	MWM
Li 2012[33]	Aβ- infused	rat	male	human	BM-MSCs	$1x10^6$	Systematic(IV)	MWM
Park& Joo 2012[34]	KA-infused	rat	male	cell line	NSCs	$1x10^6$	Stereotaxic(lateral ventricular)	PAT
Park&Lee2012[35]	AF64A cholinotoxin infused	rat	male	cell line	NSCs	$1x10^6$	Stereotaxic(lateral ventricular)	PAT
Xue 2012[36]	2×TgAD	TG mice	female	human	HAECs	$1.2x10^5$	Stereotaxic(lateral ventricular)	six-radial arm water maze test
Zhang 2012[37]	Aβ 23-35	rat	male	rat	BM-MSCs (modified)	$5X\ 10^6$	Stereotaxic(lateral ventricular)	MWM
Bobkova 2013[38]	OBE	mouse	unclear	human	MMSC	$(1-2)x10^6$	Stereotaxic(i.c.)/ Systematic(IV)	MWM
Fujiwara 2013[39]	1xTg-AD	TG mice	unclear	cell line	neuronal precursors	$2X\ 10^5$	Stereotaxic(i.h.)	MWM
Kim 2013[40]	2xTg-AD	TG mice	female/male	human	hAMSCs	$2x10^6$	Systematic(IV)	water maze test
Ma 2013[41]	2xTg-AD	TG mice	male	rat	ADMSCs	$1x10^5$	Stereotaxic(i.h.)	MWM/NORT
Park 2013[42]	old	mouse	male	cell line	NSCs (modified)	$4X\ 10^5$	Stereotaxic(lateral ventricular)	MWM PAT

Yang&Xie 2013[43]	Tg-AD	TG mice	male	human	HUMSCs (modified)	$5x10^4$	Stereotaxic(i.h.)	MWM
Yang &Yang 2013[44]	2xTg-AD	TG mice	unclear	human	UC-MSCs (modified)	$7X 10^5$	Systematic(intrac ardiac)	MWM
Yang&Yue 2013[45]	2xTg-AD	TG mice	male	human	HUMSCs	$2x10^6$	Systematic(IV)	MWM
Yun 2013[46]	Aβ- infused	mouse	male	human	PD-MSCs	$1x10^5/5x10^5$ $/1x10^6$	Systematic(IV)	MWM/PAT
Ager 2014[47]	3xTg-AD 2xTg-AD	TG mice	male/female	human	NSCs	$2X 10^5$	Stereotaxic(i.h.)	MWM NORT
Ben 2014[48]	2xTg-AD	TG mice	male	mouse	NPCs (modified)	4000	Stereotaxic(i.h.)	MMW The fear-conditioning apparatus
Chen 2014[49]	3×Tg-AD	TG mice	male	mouse	NSCs	$2x10^6$	Stereotaxic(i.h.)	MWM
Garcia 2014[50]	2xTg-AD	TG mice	male	mouse	BMSCs (modified)	$1X 10^6$	Stereotaxic(latera l ventricular)	the social recognition test(SR) PMDAT
Safar 2014[51]	Scopolamine	rat	male	rat	BM-EPCs AT-MSCs	$2X 10^6$	Systematic(IV)	Y-Maze MWM
Wang 2014[52]	Aβ 1–40	rat	Male	rat	BM-MSCs	$1X 10^5$	Stereotaxic(i.h.)	MWM
Yan 2014[53]	2xTg-AD	TG mice	unclear	rat	ADMSCs	$1x10^5$	Stereotaxic(i.h.)	NORT
Zhang&Wang 2014[54]	2xTg-AD	TG mice	unclear	mouse	NSCs (modified)	$1X 10^6$	Stereotaxic(i.h.)	MWM

Zhang&Sha 2014[55]	2xTg-AD	TG mice	unclear	mouse	NSCs (modified)	(2.5-5)X 10^6	Stereotaxic(i.h.)	MWM
Gu 2015[56]	2xTg-AD	TG mice	male	mouse	NSCs (modified)	1X 10^5	Stereotaxic(i.h.)	MWM
Marei 2015[57]	Ibo-infused	rat	male	human	OBNSC/NPCs	2.5X 10^6	Stereotaxic(i.h.)	MWM
Zhang 2015[58]	2xTg-AD	TG mice	unclear	mouse	NSCs (modified)	(2.5-5)X 10^6	Stereotaxic(i.h.)	MWM

Abbreviations: NPCs=neural precursor cells; BMSC=bone marrow stromal cells; BMSCs=Bone marrow mesenchymal stem cells; NORT= novel object recognition tasks; HAECs= human amniotic epithelial cells; BM-MSCs= bone marrow-derived mesenchymal stem cells; UC-MSCs=Human umbilical cord derived mesenchymal stem cells; BM-EPCs =Bone Marrow-Derived Endothelial Progenitor Cells; Human Olfactory Bulb Neural Stem Cells; i.h.=intra-hippocampus; IV =intravenous; i.c.= intracerebral ; nbM =nucleus basalis of Meynert; OA =okadaic acid; FF =Fimbria-fornix; TG=transgenic; Ibo= Ibotenic acid;OBE= Olfactory bulbectomized; PMDAT= plus-maze discriminative avoidance task; PAT=passive avoidance test

References

1. Wu SL, Jin LH, Li ZY, Li HL, Wang GY, Song TJ. Establishment of an animal model of Alzheimer's disease and therapeutic effects of neural stem cells on Alzheimer's disease. Chinese Journal of Clinical Rehabilitation. 2004;8(19):3734-6.

2. Wang QH, Xu RX, Nagao S. Transplantation of cholinergic neural stem cells in a mouse model of Alzheimer's disease. Chin Med J (Engl). 2005;118(6):508-11.

3. Zhao Z, Hu H, Feng G. [Learning and memory amelioration of transplantation of the neural stem cells modified with human brain-derived neurotrophic factor gene on Alzheimer disease model rat]. Chinese journal of reparative and reconstructive surgery. 2005;19(5):331-4.

4. Wang Q, Matsumoto Y, Shindo T, Miyake K, Shindo A, Kawanishi M et al. Neural stem cells transplantation in cortex in a mouse model of Alzheimer's disease. J Med Invest. 2006;53(1-2):61-9.
5. Tang J, Xu HW, Zhou GG, Fan XT, Li DB, Yang L. [Differentiation of neural precursor cells derived from mouse embryonic stem cells after transplantation into Aβ injured rat hippocampus and the improvement in memory]. Acta Academiae Medicinae Militaris Tertiae. 2007;29(02):108-11.
6. Wu QY, Li J, Feng ZT, Wang TH. Bone marrow stromal cells of transgenic mice can improve the cognitive ability of an Alzheimer's disease rat model. Neurosci Lett. 2007;417(3):281-5. doi:10.1016/j.neulet.2007.02.092.
7. Yang C, Zhang Q, Wang SC, Qiao P, Zhang Z. [Neural stem cells transplantation improved learning and memory abilities in Alzheimer's disease rat]. Chinese J Appl Physiol. 2007;23(2):159-61.
8. Gu HG, Long DH, Li XB, Zhang GP, Su T, Li JM et al. Effect of neural stem cells transplantation on parvalbumin-positive neurons of the basal forebrain and abilities of learning and memory in a rat model of senile dementia. CRTER. 2008;12(12):2235-9.
9. Li LY, Li JT, Wu QY, Li J, Feng ZT, Liu S et al. Transplantation of NGF-gene-modified bone marrow stromal cells into a rat model of Alzheimer' disease. Journal of molecular neuroscience : MN. 2008;34(2):157-63. doi:10.1007/s12031-007-9022-x.
10. Li XS, Guan FX, Li GD, Guo YN, Yang B, Du Y et al. Effects of human amnion membrane mesenchymal stem cell transplantation on behavior and beta-amyloid protein changes in transgenic mice with Alzheimer's desease. CRTER. 2008;12(51):10068-72.
11. Li ZF, Tang J, Li LS, Yang L, Yin JB, Xu HW. [Differentiation and therapeutic effects of C17.2 neural stem cells after transplanted into hippocampus of Aβ1-40-injuried rats]. Acta Academiae Medicinae Militaris Tertiae. 2008;30(7):595-9.
12. Tang J, Xu H, Fan X, Li D, Rancourt D, Zhou G et al. Embryonic stem cell-derived neural precursor cells improve memory dysfunction in Abeta(1-40) injured rats. Neurosci Res. 2008;62(2):86-96. doi:10.1016/j.neures.2008.06.005.
13. Wei CX, Chen SL. [Effects of transplantation of bone marrow mesenchymal stem cells modified by brain-derived neurotrophic factor gene on memory function of dementia rats]. CRTER 2008;12(38):7431-4.
14. Wu S, Sasaki A, Yoshimoto R, Kawahara Y, Manabe T, Kataoka K et al. Neural stem cells improve learning and memory in rats with Alzheimer's disease. Pathobiology. 2008;75(3):186-94. doi:10.1159/000124979.
15. Blurton-Jones M, Kitazawa M, Martinez-Coria H, Castello NA, Muller FJ, Loring JF et al. Neural stem cells improve cognition via BDNF in a transgenic model of Alzheimer disease. Proc Natl Acad Sci U S A. 2009;106(32):13594-9. doi:10.1073/pnas.0901402106.
16. Li HS, Chen ZF, Li HY. Effects of bone marrow mesenchymal stem cell transplantation on ethology of rats with alzheimer's disease. CRTER. 2009;13(49):9659-62.
17. Moghadam FH, Alaie H, Karbalaie K, Tanhaei S, Nasr Esfahani MH, Baharvand H. Transplantation of primed or unprimed mouse embryonic stem cell-derived neural

precursor cells improves cognitive function in Alzheimerian rats. Differentiation. 2009;78(2-3):59-68. doi:10.1016/j.diff.2009.06.005.
18. Xuan AG, Luo M, Ji WD, Long DH. Effects of engrafted neural stem cells in Alzheimer's disease rats. Neurosci Lett. 2009;450(2):167-71. doi:10.1016/j.neulet.2008.12.001.
19. He W, Bo H, Mu XH, Zhang L, Li HS. Effects of human bone marrow mesenchymal stem cell transplantation on cognitive ability and hippocampus ultrastructure in Alzheimer's disease rats. CRTER. 2010;14(40):7453-7.
20. Lee HJ, Lee JK, Lee H, Shin JW, Carter JE, Sakamoto T et al. The therapeutic potential of human umbilical cord blood-derived mesenchymal stem cells in Alzheimer's disease. Neurosci Lett. 2010;481(1):30-5. doi:10.1016/j.neulet.2010.06.045.
21. Lee JK, Jin HK, Bae JS. Bone marrow-derived mesenchymal stem cells attenuate amyloid beta-induced memory impairment and apoptosis by inhibiting neuronal cell death. Curr Alzheimer Res. 2010;7(6):540-8.
22. Lee JK, Jin HK, Endo S, Schuchman EH, Carter JE, Bae JS. Intracerebral transplantation of bone marrow-derived mesenchymal stem cells reduces amyloid-beta deposition and rescues memory deficits in Alzheimer's disease mice by modulation of immune responses. Stem Cells. 2010;28(2):329-43. doi:10.1002/stem.277.
23. Li Z, Gao C, Huang H, Sun W, Yi H, Fan X et al. Neurotransmitter phenotype differentiation and synapse formation of neural precursors engrafting in amyloid-beta(1-40) injured rat hippocampus. Journal of Alzheimer's disease : JAD. 2010;21(4):1233-47. doi:10.3233/JAD-2010-100003.
24. Pan Xb, Long Dh, Luo Xm, Tu Lg, Pan L, Wang Gp. Influence of neural stem cell transplantation on the number of p75NGFR positive neurons of the basal forebrain and the ethology of an animal model of Alzheimer's disease with 192-IgG-saporin. CRTER. 2010;14(45):8426-30.
25. Shen XL, Zhang SM, Cui MY, Jia LH. Effect of neural stem cell transplantation on cognitive function and oxidative stress in dementia rats. Progress of Anatomical Sciences. 2011;17(3):304-7.
26. Xue SR, Chen CF, Dong WL, Hui GZ, Liu TJ, Guo LH. Intracerebroventricular transplantation of human amniotic epithelial cells ameliorates spatial memory deficit in the doubly transgenic mice coexpressing APPswe and PS1DeltaE9-deleted genes. Chin Med J (Engl). 2011;124(17):2642-8.
27. Babaei P, Soltani Tehrani B, Alizadeh A. Transplanted bone marrow mesenchymal stem cells improve memory in rat models of Alzheimer's disease. Stem Cells Int. 2012;2012:369417. doi:10.1155/2012/369417.
28. Esmaeilzade B, Nobakht M, Joghataei MT, Rahbar Roshandel N, Rasouli H, Samadi Kuchaksaraei A et al. Delivery of epidermal neural crest stem cells (EPI-NCSC) to hippocamp in Alzheimer's disease rat model. Iran Biomed J. 2012;16(1):1-9. doi:10.6091/ibj.1029.2012.
29. Kim S, Chang KA, Kim J, Park HG, Ra JC, Kim HS et al. The preventive and therapeutic effects of intravenous human adipose-derived stem cells in Alzheimer's disease mice. PloS one. 2012;7(9):e45757. doi:10.1371/journal.pone.0045757.
30. Lee HJ, Lee JK, Lee H, Carter JE, Chang JW, Oh W et al. Human umbilical cord blood-derived mesenchymal stem cells improve neuropathology and cognitive impairment in an Alzheimer's disease mouse model through modulation of neuroinflammation. Neurobiol Aging. 2012;33(3):588-602. doi:10.1016/j.neurobiolaging.2010.03.024.

31. Lee HJ, Lim IJ, Park SW, Kim YB, Ko Y, Kim SU. Human neural stem cells genetically modified to express human nerve growth factor (NGF) gene restore cognition in the mouse with ibotenic acid-induced cognitive dysfunction. Cell transplantation. 2012;21(11):2487-96. doi:10.3727/096368912X638964.
32. Lee JK, Schuchman EH, Jin HK, Bae JS. Soluble CCL5 derived from bone marrow-derived mesenchymal stem cells and activated by amyloid beta ameliorates Alzheimer's disease in mice by recruiting bone marrow-induced microglia immune responses. Stem Cells. 2012;30(7):1544-55. doi:10.1002/stem.1125.
33. Li WY, Jin RL, Hu XY. [Migration of PKH26-labeled mesenchymal stem cells in rats with Alzheimer's disease]. Journal of Zhejiang University Medical sciences. 2012;41(6):659-64.
34. Park D, Joo SS, Kim TK, Lee SH, Kang H, Lee HJ et al. Human neural stem cells overexpressing choline acetyltransferase restore cognitive function of kainic acid-induced learning and memory deficit animals. Cell transplantation. 2012;21(1):365-71. doi:10.3727/096368911X586765.
35. Park D, Lee HJ, Joo SS, Bae DK, Yang G, Yang YH et al. Human neural stem cells over-expressing choline acetyltransferase restore cognition in rat model of cognitive dysfunction. Experimental neurology. 2012;234(2):521-6. doi:10.1016/j.expneurol.2011.12.040.
36. Xue S, Chen C, Dong W, Hui G, Liu T, Guo L. Therapeutic effects of human amniotic epithelial cell transplantation on double-transgenic mice co-expressing APPswe and PS1DeltaE9-deleted genes. Science China Life sciences. 2012;55(2):132-40. doi:10.1007/s11427-012-4283-1.
37. Zhang P, Zhao G, Kang X, Su L. Effects of lateral ventricular transplantation of bone marrow-derived mesenchymal stem cells modified with brain-derived neurotrophic factor gene on cognition in a rat model of Alzheimer's disease. Neural regeneration research. 2012;7(4):245-50. doi:10.3969/j.issn.1673-5374.2012.04.001.
38. Bobkova NV, Poltavtseva RA, Samokhin AN, Sukhikh GT. Therapeutic effect of mesenchymal multipotent stromal cells on memory in animals with Alzheimer-type neurodegeneration. Bulletin of experimental biology and medicine. 2013;156(1):119-21.
39. Fujiwara N, Shimizu J, Takai K, Arimitsu N, Saito A, Kono T et al. Restoration of spatial memory dysfunction of human APP transgenic mice by transplantation of neuronal precursors derived from human iPS cells. Neurosci Lett. 2013. doi:10.1016/j.neulet.2013.10.043.
40. Kim KS, Kim HS, Park JM, Kim HW, Park MK, Lee HS et al. Long-term immunomodulatory effect of amniotic stem cells in an Alzheimer's disease model. Neurobiol Aging. 2013;34(10):2408-20. doi:10.1016/j.neurobiolaging.2013.03.029.
41. Ma T, Gong K, Ao Q, Yan Y, Song B, Huang H et al. Intracerebral transplantation of adipose-derived mesenchymal stem cells alternatively activates microglia and ameliorates neuropathological deficits in Alzheimer's disease mice. Cell transplantation. 2013;22 Suppl 1(1):S113-26. doi:10.3727/096368913X672181.
42. Park D, Yang YH, Bae DK, Lee SH, Yang G, Kyung J et al. Improvement of cognitive function and physical activity of aging mice by human neural stem cells over-expressing choline acetyltransferase. Neurobiol Aging. 2013;34(11):2639-46. doi:10.1016/j.neurobiolaging.2013.04.026.
43. Yang H, Xie Z, Wei L, Yang H, Yang S, Zhu Z et al. Human umbilical cord mesenchymal stem cell-derived neuron-like cells rescue memory deficits and reduce amyloid-beta deposition in an AbetaPP/PS1 transgenic mouse model. Stem Cell Res Ther. 2013;4(4):76. doi:10.1186/scrt227.

44. Yang H, Yang H, Xie Z, Wei L, Bi J. Systemic transplantation of human umbilical cord derived mesenchymal stem cells-educated T regulatory cells improved the impaired cognition in AbetaPPswe/PS1dE9 transgenic mice. PloS one. 2013;8(7):e69129. doi:10.1371/journal.pone.0069129.
45. Yang H, Yue C, Yang H, Xie Z, Hu H, Wei L et al. Intravenous Administration of Human Umbilical Cord Mesenchymal Stem Cells Improves Cognitive Impairments and Reduces Amyloid-Beta Deposition in an AbetaPP/PS1 Transgenic Mouse Model. Neurochem Res. 2013;38(12):2474-82. doi:10.1007/s11064-013-1161-6.
46. Yun HM, Kim HS, Park KR, Shin JM, Kang AR, il Lee K et al. Placenta-derived mesenchymal stem cells improve memory dysfunction in an Abeta1-42-infused mouse model of Alzheimer's disease. Cell death & disease. 2013;4(12):e958. doi:10.1038/cddis.2013.490.
47. Ager RR, Davis JL, Agazaryan A, Benavente F, Poon WW, LaFerla FM et al. Human neural stem cells improve cognition and promote synaptic growth in two complementary transgenic models of Alzheimer's disease and neuronal loss. Hippocampus. 2014. doi:10.1002/hipo.22405.
48. Ben-Menachem-Zidon O, Ben-Menahem Y, Ben-Hur T, Yirmiya R. Intra-hippocampal transplantation of neural precursor cells with transgenic over-expression of IL-1 receptor antagonist rescues memory and neurogenesis impairments in an Alzheimer's disease model. Neuropsychopharmacology. 2014;39(2):401-14. doi:10.1038/npp.2013.208.
49. Chen SQ, Cai Q, Shen YY, Wang PY, Li MH, Teng GY. Neural stem cell transplantation improves spatial learning and memory via neuronal regeneration in amyloid-beta precursor protein/presenilin 1/tau triple transgenic mice. Am J Alzheimers Dis Other Demen. 2014;29(2):142-9. doi:10.1177/1533317513506776.
50. Garcia KO, Ornellas FL, Martin PK, Patti CL, Mello LE, Frussa-Filho R et al. Therapeutic effects of the transplantation of VEGF overexpressing bone marrow mesenchymal stem cells in the hippocampus of murine model of Alzheimer's disease. Front Aging Neurosci. 2014;6:30. doi:10.3389/fnagi.2014.00030.
51. Safar MM, Arab HH, Rizk SM, El-Maraghy SA. Bone Marrow-Derived Endothelial Progenitor Cells Protect Against Scopolamine-Induced Alzheimer-Like Pathological Aberrations. Mol Neurobiol. 2014. doi:10.1007/s12035-014-9051-8.
52. Wang Z, Ren XQ, Wei DX. [Hippocampus transplantation of bone marrow mesenchymal stem cells improves the memory function of Alzheimer's disease rats]. CRTER. 2014;18(50):8130-4. doi:10.3969/j.issn.2095-4344.
53. Yan Y, Ma T, Gong K, Ao Q, Zhang X, Gong Y. Adipose-derived mesenchymal stem cell transplantation promotes adult neurogenesis in the brains of Alzheimer's disease mice. Neural regeneration research. 2014;9(8):798-805. doi:10.4103/1673-5374.131596.
54. Zhang W, Wang GM, Wang PJ, Zhang Q, Sha SH. Effects of neural stem cells on synaptic proteins and memory in a mouse model of Alzheimer's disease. J Neurosci Res. 2014;92(2):185-94. doi:10.1002/jnr.23299.
55. Zhang W, Wang PJ, Sha HY, Ni J, Li MH, Gu GJ. Neural stem cell transplants improve cognitive function without altering amyloid pathology in an APP/PS1 double transgenic model of Alzheimer's disease. Mol Neurobiol. 2014;50(2):423-37. doi:10.1007/s12035-014-8640-x.
56. Gu G, Zhang W, Li M, Ni J, Wang P. Transplantation of NSC-derived cholinergic neuron-like cells improves cognitive function in APP/PS1 transgenic mice. Neuroscience.

2015;291:81-92. doi:10.1016/j.neuroscience.2015.01.073.
57. Marei HE, Farag A, Althani A, Afifi N, Abd-Elmaksoud A, Lashen S et al. Human olfactory bulb neural stem cells expressing hNGF restore cognitive deficit in Alzheimer's disease rat model. J Cell Physiol. 2015;230(1):116-30. doi:10.1002/jcp.24688.
58. Zhang W, Gu GJ, Shen X, Zhang Q, Wang GM, Wang PJ. Neural stem cell transplantation enhances mitochondrial biogenesis in a transgenic mouse model of Alzheimer's disease-like pathology. Neurobiol Aging. 2015;36(3):1282-92. doi:10.1016/j.neurobiolaging.2014.10.040.
