# Supplementary material for: Effects of stem cell transplantation on cognitive decline in animal models of Alzheimer’s disease: A systematic review and meta-analysis
Source: Sci Rep. 2015 Jul 10;5:12134. doi: 10.1038/srep12134 (PMC4498325; doi:10.1038/srep12134)
Supplement: Supplementary Information [file srep12134-s2.pdf]

Effects of stem cell transplantation on cognitive decline in animal models of Alzheimer's disease: A systematic review and meta-analysis

Zhe Wang,MD, Weijun Peng,MD, Chunhu Zhang, PhD, Chenxia Sheng, PhD, Wei Huang, PhD, Yang Wang, PhD , Rong Fan, PhD

Table S2 Quality assessment of the included studies

	(1)	(2)	(3)				(4)	(5)	(6)	(7)	(8)	(9)	(10)	(11)	(12)	(13)	(14)	(15)	(16)	(17)	Quality score
			a	b	c	d															
Wu 2004[1]	√	√	√	√	√	√	√	√	√	√	?	?	√	√	√	?	?	?	√	?	14
Wang 2005[2]	√	√	√	√	√	√	√	√	?	√	?	?	√	√	√	?	?	?	√	?	13
Zhao 2005[3]	√	√	√	√	√	?	√	√	√	√	?	?	√	√	√	√	?	?	√	?	14
Wang2006[4]	√	√	√	√	√	√	√	√	?	√	?	?	√	√	√	?	?	?	√	?	13
Tang 2007[5]	√	√	√	√	√	?	√	√	?	√	?	?	√	√	√	?	?	?	√	?	12
Wu 2007[6]	√	√	√	√	√	?	√	√	?	√	?	?	√	√	√	?	?	?	?	?	11
Yang 2007[7]	√	√	√	√	√	√	√	√	√	√	?	?	√	√	√	?	?	?	√	?	14
Gu 2008[8]	√	√	√	√	√	√	√	√	√	√	?	?	√	√	√	?	?	?	√	?	14
Li 2008a[9]	√	√	√	√	√	?	√	√	√	√	?	?	√	√	√	?	?	?	√	?	13
Li 2008b[10]	√	√	√	√	√	√	√	√	√	√	?	?	√	√	√	√	?	?	√	?	15
Li 2008c[11]	√	√	√	√	√	√	√	√	?	√	?	?	√	√	√	?	?	?	√	?	13
Tang 2008[12]	√	√	√	√	√	√	√	√	?	√	?	?	√	√	√	?	?	?	√	?	13
Wei 2008[13]	√	√	√	√	√	√	√	√	√	√	?	?	√	√	√	?	?	?	√	?	14

Wu 2008[14]	√	√	√	√	√	?	√	√	?	√	?	?	√	√	√	?	?	?	√	?	12
Blurton-Jones 2009[15]	√	√	√	√	?	?	√	√	?	√	?	?	√	√	√	?	√	?	?	?	10
Li 2009[16]	√	√	√	√	√	√	√	√	√	√	?	?	√	√	√	√	?	?	√	?	15
Moghadam 2009[17]	√	√	√	√	√	?	√	√	√	√	?	?	√	√	√	?	?	?	√	?	13
Xuan 2009[18]	√	√	√	√	√	√	√	√	√	√	?	?	√	√	√	?	?	?	√	?	14
He 2010[19]	√	√	√	√	√	√	√	√	√	√	?	?	√	√	√	√	?	?	√	?	15
Lee 2010a[20]	√	√	√	√	?	√	√	√	?	√	?	?	√	√	√	?	?	?	√	?	12
Lee 2010b[21]	√	√	√	√	?	?	√	√	?	√	?	?	√	√	√	?	?	?	√	?	11
Lee 2010c[22]	√	√	√	√	√	?	√	√	?	√	√	√	√	√	√	?	?	?	√	?	14
Li 2010[23]	√	√	√	√	√	√	√	√	?	√	?	?	√	√	√	?	?	?	√	?	13
Pan 2010[24]	√	√	√	√	√	√	√	√	√	√	?	?	√	√	√	√	?	?	√	?	15
Shen 2011[25]	√	√	√	√	√	√	√	√	√	√	?	?	√	√	√	?	?	?	√	?	14
Xue 2011[26]	√	√	√	√	√	?	√	√	√	√	?	?	√	√	√	?	?	?	√	?	13
Babaei 2012[27]	√	√	√	√	√	√	√	√	?	√	?	?	√	√	√	?	?	?	√	?	13
Esmaeilzade 2012[28]	√	√	√	√	√	?	√	√	√	√	?	?	√	√	√	?	?	?	√	?	13
Kim 2012[29]	√	√	√	√	√	√	√	√	?	√	√	√	√	√	√	?	?	?	?	?	14

Lee 2012a [30]	√	√	√	√	√	?	√	√	?	√	√	√	√	√	√	?	?	?	√	?	14
Lee2012b[31]	√	√	√	√	√	?	√	√	√	√	?	?	√	√	√	?	?	?	√	?	13
Lee2012c[32]	√	√	√	√	?	√	√	√	?	√	?	?	√	√	√	?	?	?	√	?	12
Li 2012[33]	√	√	√	√	√	√	√	√	√	√	√	?	√	√	√	?	?	?	√	?	15
Park2012a[34]	√	√	√	√	√	?	√	√	?	√	?	?	√	√	√	?	?	?	√	?	12
Park2012b[35]	√	√	√	√	√	?	√	√	?	√	?	?	√	√	√	?	?	?	√	?	12
Xue 2012[36]	√	√	√	√	?	√	√	√	√	√	?	?	√	√	√	?	?	?	√	√	14
Zhang2012[37]	√	√	√	√	√	?	√	√	√	√	?	?	√	√	√	√	?	?	√	?	14
Bobkova 2013 [38]	√	√	√	√	?	√	√	√	?	√	?	?	√	√	√	?	?	?	?	?	11
Fujiwara 2013[39]	√	√	√	√	?	?	√	√	?	√	?	?	√	√	√	?	?	?	√	?	11
Kim 2013[40]	√	√	√	√	√	√	√	√	?	√	?	?	√	√	√	?	?	?	√	√	14
Ma 2013[41]	√	√	√	√	√	√	√	√	?	√	√	?	√	√	√	?	?	?	√	?	14
Park 2013[42]	√	√	√	√	√	√	√	√	?	√	?	?	√	√	√	?	?	?	√	?	13
Yang 2013a[43]	√	√	√	√	√	√	√	√	?	√	√	√	√	√	√	?	√	?	√	?	16
Yang 2013b[44]	√	√	√	√	?	?	√	√	?	√	?	?	√	√	√	?	?	?	√	?	11
Yang 2013c[45]	√	√	√	√	√	√	√	√	√	√	?	√	√	√	√	√	√	√	√	?	18
Yun2013[46]	√	√	√	√	√	√	√	√	?	√	?	?	√	√	√	?	?	?	√	?	13

Ager 2014[47]	√	√	√	√	√	?	√	√	?	√	?	?	√	√	√	?	?	?	?	?	11
Ben 2014[48]	√	√	√	√	√	√	√	√	?	√	√	?	√	√	√	?	?	?	√	?	14
Chen 2014[49]	√	√	√	√	√	√	√	√	√	√	√	?	√	√	√	?	?	?	√	?	15
Garcia 2014[50]	√	√	√	√	√	?	√	√	?	√	√	?	√	√	√	?	?	?	√	?	13
Safar 2014[51]	√	√	√	√	√	?	√	√	√	√	?	√	√	√	√	?	?	?	?	?	13
Wang2014[52]	√	√	√	√	√	√	√	√	√	√	?	?	√	√	√	√	?	?	√	?	15
Yan2014[53]	√	√	√	√	?	√	√	√	√	√	√	?	√	√	√	?	?	?	√	?	14
Zhang2014a[54]	√	√	√	√	?	√	√	√	?	√	?	?	√	√	√	?	?	?	√	?	12
Zhang2014b[55]	√	√	√	√	?	√	√	√	?	√	?	?	√	√	√	?	?	?	√	?	12
Gu 2015[56]	√	√	√	√	√	√	√	√	√	√	?	?	√	√	√	?	?	?	√	?	14
Marei 2015[57]	√	√	√	√	√	?	√	√	?	√	?	?	√	√	√	?	?	?	?	?	11
Zhang 2015[58]	√	√	√	√	?	√	√	√	√	√	?	?	√	√	√	?	?	?	√	?	13

Note:(1) Was the research question specified and clear? (2) Were the outcome measures relevant for AD research? (3) Are the characteristics [(a) Species(b) Background(c) Sex (d) Age] of the study population clear? (4) Was the correct control group present? (5) Were the groups similar at baseline (if not randomized, were they similar with respect to characteristics such as weight and sex)? (6) Was the experiment randomized? (7) Was the type of stem cell(s) mentioned? (8) What was the age at which stem cell transplantation was started? (9) Was the duration of stem cell transplantation clear and specified? (10) Was the number of stem cells mentioned? (11) Was the administration route specified? (12) Were the methods used for outcome assessment the same in both groups? (13) Were the dropouts described for each group separately? (14) Was the outcome assessment blinded? (15) Was the outcome assessment randomized across the groups? (16) Was the total number of animals included in the statistical analyses clear? (17) Was the age at which the animals were sacrificed mentioned?

References

1. Hong SQ, Zhang HT, You J, Zhang MY, Cai YQ, Jiang XD et al. Comparison of transdifferentiated and untransdifferentiated human umbilical mesenchymal stem cells in rats after traumatic brain injury. Neurochem Res. 2011;36(12):2391-400. doi:10.1007/s11064-011-0567-2.

2. Wang QH, Xu RX, Nagao S. Transplantation of cholinergic neural stem cells in a mouse model of Alzheimer's disease. Chin Med J (Engl). 2005;118(6):508-11.

3. Zhao Z, Hu H, Feng G. [Learning and memory amelioration of transplantation of the neural stem cells modified with human brain-derived neurotrophic factor gene on Alzheimer disease model rat]. Chinese journal of reparative and reconstructive surgery. 2005;19(5):331-4.

4. Wang Q, Matsumoto Y, Shindo T, Miyake K, Shindo A, Kawanishi M et al. Neural stem cells transplantation in cortex in a mouse model of Alzheimer's disease. J Med Invest. 2006;53(1-2):61-9.

5. Tang J, Xu HW, Zhou GG, Fan XT, Li DB, Yang L. [Differentiation of neural precursor cells derived from mouse embryonic stem cells after transplantation into Aβ injured rat hippocampus and the improvement in memory]. Acta Academiae Medicinae Militaris Tertiae. 2007;29(02):108-11.

6. Wu QY, Li J, Feng ZT, Wang TH. Bone marrow stromal cells of transgenic mice can improve the cognitive ability of an Alzheimer's disease rat model. Neurosci Lett. 2007;417(3):281-5. doi:10.1016/j.neulet.2007.02.092.

7. Yang C, Zhang Q, Wang SC, Qiao P, Zhang Z. [Neural stem cells transplantation improved learning and memory abilities in Alzheimer's disease rat]. Chinese J Appl Physiol. 2007;23(2):159-61.

8. Gu HG, Long DH, Li XB, Zhang GP, Su T, Li JM et al. Effect of neural stem cells transplantation on parvalbumin-positive neurons of the basal forebrain and abilities of learning and memory in a rat model of senile dementia. CRTER. 2008;12(12):2235-9.

9. Li LY, Li JT, Wu QY, Li J, Feng ZT, Liu S et al. Transplantation of NGF-gene-modified bone marrow stromal cells into a rat model of Alzheimer' disease. Journal of molecular neuroscience : MN. 2008;34(2):157-63. doi:10.1007/s12031-007-9022-x.

10. Li XS, Guan FX, Li GD, Guo YN, Yang B, Du Y et al. Effects of human amnion membrane mesenchymal stem cell transplantation on behavior and beta-amyloid protein changes in transgenic mice with Alzheimer's desease. CRTER. 2008;12(51):10068-72.

11. Li ZF, Tang J, Li LS, Yang L, Yin JB, Xu HW. [Differentiation and therapeutic effects of C17.2 neural stem cells after transplanted into hippocampus of Aβ1-40-injuried rats].

Acta Academiae Medicinae Militaris Tertiae. 2008;30(7):595-9.
12. Tang J, Xu H, Fan X, Li D, Rancourt D, Zhou G et al. Embryonic stem cell-derived neural precursor cells improve memory dysfunction in Abeta(1-40) injured rats. Neurosci Res. 2008;62(2):86-96. doi:10.1016/j.neures.2008.06.005.
13. Wei CX, Chen SL. [Effects of transplantation of bone marrow mesenchymal stem cells modified by brain-derived neurotrophic factor gene on memory function of dementia rats]. CRTER 2008;12(38):7431-4.
14. Wu S, Sasaki A, Yoshimoto R, Kawahara Y, Manabe T, Kataoka K et al. Neural stem cells improve learning and memory in rats with Alzheimer's disease. Pathobiology. 2008;75(3):186-94. doi:10.1159/000124979.
15. Blurton-Jones M, Kitazawa M, Martinez-Coria H, Castello NA, Muller FJ, Loring JF et al. Neural stem cells improve cognition via BDNF in a transgenic model of Alzheimer disease. Proc Natl Acad Sci U S A. 2009;106(32):13594-9. doi:10.1073/pnas.0901402106.
16. Li HS, Chen ZF, Li HY. Effects of bone marrow mesenchymal stem cell transplantation on ethology of rats with alzheimer's disease. CRTER. 2009;13(49):9659-62.
17. Moghadam FH, Alaie H, Karbalaie K, Tanhaei S, Nasr Esfahani MH, Baharvand H. Transplantation of primed or unprimed mouse embryonic stem cell-derived neural precursor cells improves cognitive function in Alzheimerian rats. Differentiation. 2009;78(2-3):59-68. doi:10.1016/j.diff.2009.06.005.
18. Xuan AG, Luo M, Ji WD, Long DH. Effects of engrafted neural stem cells in Alzheimer's disease rats. Neurosci Lett. 2009;450(2):167-71. doi:10.1016/j.neulet.2008.12.001.
19. He W, Bo H, Mu XH, Zhang L, Li HS. Effects of human bone marrow mesenchymal stem cell transplantation on cognitive ability and hippocampus ultrastructure in Alzheimer's disease rats. CRTER. 2010;14(40):7453-7.
20. Lee HJ, Lee JK, Lee H, Shin JW, Carter JE, Sakamoto T et al. The therapeutic potential of human umbilical cord blood-derived mesenchymal stem cells in Alzheimer's disease. Neurosci Lett. 2010;481(1):30-5. doi:10.1016/j.neulet.2010.06.045.
21. Lee JK, Jin HK, Bae JS. Bone marrow-derived mesenchymal stem cells attenuate amyloid beta-induced memory impairment and apoptosis by inhibiting neuronal cell death. Curr Alzheimer Res. 2010;7(6):540-8.
22. Lee JK, Jin HK, Endo S, Schuchman EH, Carter JE, Bae JS. Intracerebral transplantation of bone marrow-derived mesenchymal stem cells reduces amyloid-beta deposition and rescues memory deficits in Alzheimer's disease mice by modulation of immune responses. Stem Cells. 2010;28(2):329-43. doi:10.1002/stem.277.
23. Li Z, Gao C, Huang H, Sun W, Yi H, Fan X et al. Neurotransmitter phenotype differentiation and synapse formation of neural precursors engrafting in amyloid-beta(1-40) injured rat hippocampus. Journal of Alzheimer's disease : JAD. 2010;21(4):1233-47. doi:10.3233/JAD-2010-100003.
24. Pan Xb, Long Dh, Luo Xm, Tu Lg, Pan L, Wang Gp. Influence of neural stem cell transplantation on the number of p75NGFR positive neurons of the basal forebrain and the ethology of an animal model of Alzheimer's disease with 192-IgG-saporin. CRTER. 2010;14(45):8426-30.
25. Shen XL, Zhang SM, Cui MY, Jia LH. Effect of neural stem cell transplantation on cognitive function and oxidative stress in dementia rats. Progress of Anatomical Sciences.

2011;17(3):304-7.
26. Xue SR, Chen CF, Dong WL, Hui GZ, Liu TJ, Guo LH. Intracerebroventricular transplantation of human amniotic epithelial cells ameliorates spatial memory deficit in the doubly transgenic mice coexpressing APPswe and PS1DeltaE9-deleted genes. Chin Med J (Engl). 2011;124(17):2642-8.
27. Babaei P, Soltani Tehrani B, Alizadeh A. Transplanted bone marrow mesenchymal stem cells improve memory in rat models of Alzheimer's disease. Stem Cells Int. 2012;2012:369417. doi:10.1155/2012/369417.
28. Esmaeilzade B, Nobakht M, Joghataei MT, Rahbar Roshandel N, Rasouli H, Samadi Kuchaksaraei A et al. Delivery of epidermal neural crest stem cells (EPI-NCSC) to hippocamp in Alzheimer's disease rat model. Iran Biomed J. 2012;16(1):1-9. doi:10.6091/ibj.1029.2012.
29. Kim S, Chang KA, Kim J, Park HG, Ra JC, Kim HS et al. The preventive and therapeutic effects of intravenous human adipose-derived stem cells in Alzheimer's disease mice. PloS one. 2012;7(9):e45757. doi:10.1371/journal.pone.0045757.
30. Lee HJ, Lee JK, Lee H, Carter JE, Chang JW, Oh W et al. Human umbilical cord blood-derived mesenchymal stem cells improve neuropathology and cognitive impairment in an Alzheimer's disease mouse model through modulation of neuroinflammation. Neurobiol Aging. 2012;33(3):588-602. doi:10.1016/j.neurobiolaging.2010.03.024.
31. Lee HJ, Lim IJ, Park SW, Kim YB, Ko Y, Kim SU. Human neural stem cells genetically modified to express human nerve growth factor (NGF) gene restore cognition in the mouse with ibotenic acid-induced cognitive dysfunction. Cell transplantation. 2012;21(11):2487-96. doi:10.3727/096368912X638964.
32. Lee JK, Schuchman EH, Jin HK, Bae JS. Soluble CCL5 derived from bone marrow-derived mesenchymal stem cells and activated by amyloid beta ameliorates Alzheimer's disease in mice by recruiting bone marrow-induced microglia immune responses. Stem Cells. 2012;30(7):1544-55. doi:10.1002/stem.1125.
33. Li WY, Jin RL, Hu XY. [Migration of PKH26-labeled mesenchymal stem cells in rats with Alzheimer's disease]. Journal of Zhejiang University Medical sciences. 2012;41(6):659-64.
34. Park D, Joo SS, Kim TK, Lee SH, Kang H, Lee HJ et al. Human neural stem cells overexpressing choline acetyltransferase restore cognitive function of kainic acid-induced learning and memory deficit animals. Cell transplantation. 2012;21(1):365-71. doi:10.3727/096368911X586765.
35. Park D, Lee HJ, Joo SS, Bae DK, Yang G, Yang YH et al. Human neural stem cells over-expressing choline acetyltransferase restore cognition in rat model of cognitive dysfunction. Experimental neurology. 2012;234(2):521-6. doi:10.1016/j.expneurol.2011.12.040.
36. Xue S, Chen C, Dong W, Hui G, Liu T, Guo L. Therapeutic effects of human amniotic epithelial cell transplantation on double-transgenic mice co-expressing APPswe and PS1DeltaE9-deleted genes. Science China Life sciences. 2012;55(2):132-40. doi:10.1007/s11427-012-4283-1.
37. Zhang P, Zhao G, Kang X, Su L. Effects of lateral ventricular transplantation of bone marrow-derived mesenchymal stem cells modified with brain-derived neurotrophic factor gene on cognition in a rat model of Alzheimer's disease. Neural regeneration research. 2012;7(4):245-50. doi:10.3969/j.issn.1673-5374.2012.04.001.
38. Bobkova NV, Poltavtseva RA, Samokhin AN, Sukhikh GT. Therapeutic effect of mesenchymal multipotent stromal cells on memory in animals with Alzheimer-type

neurodegeneration. Bulletin of experimental biology and medicine. 2013;156(1):119-21.
39. Fujiwara N, Shimizu J, Takai K, Arimitsu N, Saito A, Kono T et al. Restoration of spatial memory dysfunction of human APP transgenic mice by transplantation of neuronal precursors derived from human iPS cells. Neurosci Lett. 2013. doi:10.1016/j.neulet.2013.10.043.
40. Kim KS, Kim HS, Park JM, Kim HW, Park MK, Lee HS et al. Long-term immunomodulatory effect of amniotic stem cells in an Alzheimer's disease model. Neurobiol Aging. 2013;34(10):2408-20. doi:10.1016/j.neurobiolaging.2013.03.029.
41. Ma T, Gong K, Ao Q, Yan Y, Song B, Huang H et al. Intracerebral transplantation of adipose-derived mesenchymal stem cells alternatively activates microglia and ameliorates neuropathological deficits in Alzheimer's disease mice. Cell transplantation. 2013;22 Suppl 1(1):S113-26. doi:10.3727/096368913X672181.
42. Park D, Yang YH, Bae DK, Lee SH, Yang G, Kyung J et al. Improvement of cognitive function and physical activity of aging mice by human neural stem cells over-expressing choline acetyltransferase. Neurobiol Aging. 2013;34(11):2639-46. doi:10.1016/j.neurobiolaging.2013.04.026.
43. Yang H, Xie Z, Wei L, Yang H, Yang S, Zhu Z et al. Human umbilical cord mesenchymal stem cell-derived neuron-like cells rescue memory deficits and reduce amyloid-beta deposition in an AbetaPP/PS1 transgenic mouse model. Stem Cell Res Ther. 2013;4(4):76. doi:10.1186/scrt227.
44. Yang H, Yang H, Xie Z, Wei L, Bi J. Systemic transplantation of human umbilical cord derived mesenchymal stem cells-educated T regulatory cells improved the impaired cognition in AbetaPPswe/PS1dE9 transgenic mice. PloS one. 2013;8(7):e69129. doi:10.1371/journal.pone.0069129.
45. Yang H, Yue C, Yang H, Xie Z, Hu H, Wei L et al. Intravenous Administration of Human Umbilical Cord Mesenchymal Stem Cells Improves Cognitive Impairments and Reduces Amyloid-Beta Deposition in an AbetaPP/PS1 Transgenic Mouse Model. Neurochem Res. 2013;38(12):2474-82. doi:10.1007/s11064-013-1161-6.
46. Yun HM, Kim HS, Park KR, Shin JM, Kang AR, il Lee K et al. Placenta-derived mesenchymal stem cells improve memory dysfunction in an Abeta1-42-infused mouse model of Alzheimer's disease. Cell death & disease. 2013;4(12):e958. doi:10.1038/cddis.2013.490.
47. Ager RR, Davis JL, Agazaryan A, Benavente F, Poon WW, LaFerla FM et al. Human neural stem cells improve cognition and promote synaptic growth in two complementary transgenic models of Alzheimer's disease and neuronal loss. Hippocampus. 2014. doi:10.1002/hipo.22405.
48. Ben-Menachem-Zidon O, Ben-Menahem Y, Ben-Hur T, Yirmiya R. Intra-hippocampal transplantation of neural precursor cells with transgenic over-expression of IL-1 receptor antagonist rescues memory and neurogenesis impairments in an Alzheimer's disease model. Neuropsychopharmacology. 2014;39(2):401-14. doi:10.1038/npp.2013.208.
49. Chen SQ, Cai Q, Shen YY, Wang PY, Li MH, Teng GY. Neural stem cell transplantation improves spatial learning and memory via neuronal regeneration in amyloid-beta precursor protein/presenilin 1/tau triple transgenic mice. Am J Alzheimers Dis Other Demen. 2014;29(2):142-9. doi:10.1177/1533317513506776.
50. Garcia KO, Ornellas FL, Martin PK, Patti CL, Mello LE, Frussa-Filho R et al. Therapeutic effects of the transplantation of VEGF overexpressing bone marrow mesenchymal stem cells in the hippocampus of murine model of Alzheimer's disease. Front Aging Neurosci. 2014;6:30. doi:10.3389/fnagi.2014.00030.

51. Safar MM, Arab HH, Rizk SM, El-Maraghy SA. Bone Marrow-Derived Endothelial Progenitor Cells Protect Against Scopolamine-Induced Alzheimer-Like Pathological Aberrations. Mol Neurobiol. 2014. doi:10.1007/s12035-014-9051-8.
52. Wang Z, Ren XQ, Wei DX. [Hippocampus transplantation of bone marrow mesenchymal stem cells improves the memory function of Alzheimer's disease rats]. CRTER. 2014;18(50):8130-4. doi:10.3969/j.issn.2095-4344.
53. Yan Y, Ma T, Gong K, Ao Q, Zhang X, Gong Y. Adipose-derived mesenchymal stem cell transplantation promotes adult neurogenesis in the brains of Alzheimer's disease mice. Neural regeneration research. 2014;9(8):798-805. doi:10.4103/1673-5374.131596.
54. Zhang W, Wang GM, Wang PJ, Zhang Q, Sha SH. Effects of neural stem cells on synaptic proteins and memory in a mouse model of Alzheimer's disease. J Neurosci Res. 2014;92(2):185-94. doi:10.1002/jnr.23299.
55. Zhang W, Wang PJ, Sha HY, Ni J, Li MH, Gu GJ. Neural stem cell transplants improve cognitive function without altering amyloid pathology in an APP/PS1 double transgenic model of Alzheimer's disease. Mol Neurobiol. 2014;50(2):423-37. doi:10.1007/s12035-014-8640-x.
56. Gu G, Zhang W, Li M, Ni J, Wang P. Transplantation of NSC-derived cholinergic neuron-like cells improves cognitive function in APP/PS1 transgenic mice. Neuroscience. 2015;291:81-92. doi:10.1016/j.neuroscience.2015.01.073.
57. Marei HE, Farag A, Althani A, Afifi N, Abd-Elmaksoud A, Lashen S et al. Human olfactory bulb neural stem cells expressing hNGF restore cognitive deficit in Alzheimer's disease rat model. J Cell Physiol. 2015;230(1):116-30. doi:10.1002/jcp.24688.
58. Zhang W, Gu GJ, Shen X, Zhang Q, Wang GM, Wang PJ. Neural stem cell transplantation enhances mitochondrial biogenesis in a transgenic mouse model of Alzheimer's disease-like pathology. Neurobiol Aging. 2015;36(3):1282-92. doi:10.1016/j.neurobiolaging.2014.10.040.
