# Supplementary material for: Effects of stem cell transplantation on cognitive decline in animal models of Alzheimer’s disease: A systematic review and meta-analysis
Source: Sci Rep. 2015 Jul 10;5:12134. doi: 10.1038/srep12134 (PMC4498325; doi:10.1038/srep12134)
Supplement: Supplementary Information [file srep12134-s3.pdf]

Effects of stem cell transplantation on cognitive decline in animal models of Alzheimer's disease: A systematic review and meta-analysis

Zhe Wang,MD, Weijun Peng,MD, Chunhu Zhang, PhD, Chenxia Sheng, PhD, Wei Huang, PhD, Yang Wang, PhD , Rong Fan, PhD

Tables S3 Study characteristics accounting for heterogeneity

3.1 Learning function: Stratified analysis of stem cell-treated *vs*. control

	Number of comparisons	Number of Participants	Std.Mean difference (IV, random,95%CI)	*P*	Adjusted R^2
Pooled estimate	52	1045	-1.47 [-1.74, -1.20]		
AD model					
Transgenic	20	472	-1.14 [-1.48, -0.80]	0.10	N/A
Aβ- infused	15	278	-1.97 [-2.67, -1.28]		
F-F transaction	4	75	-1.98 [-2.83, -1.13]		
Chem- infused	10	168	-1.58 [-2.32, -0.84]		
Natural senile	3	52	-1.10 [-1.70, -0.50]		
Recipient species					
TG	18	432	-1.20 [-1.57, -0.82]	0.02	3.72%
Rat	23	406	-2.02 [-2.59, -1.44]		
Mouse	11	207	-1.09 [-1.40, -0.77]		
Recipient sex					
Both	1	19	-1.41 [-2.44, -0.38]	0.008	N/A

Male	40	786	-1.71 [-2.07, -1.34]		
Female	2	36	-1.06 [-2.05, -0.08]		
Unclear	9	204	-0.90 [-1.19, -0.60]		
Donor species					
Human	13	318	-1.07 [-1.42, -0.73]		
Mouse	11	236	-1.36 [-1.94, -0.77]		
Rats	16	293	-2.24 [-2.97, -1.50]	0.05	N/A
Cell line	12	198	-1.33 [-1.82, -0.85]		
Type of stem cells					
MSCs	22	480	-1.29 [-1.66, -0.92]		
NSCs	22	427	-1.60 [-2.07, -1.13]		
NPCs	6	87	-2.08 [-3.23, -0.93]	0.38	N/A
Other	2	51	-1.06 [-1.84, -0.27]		
Number of cells injected					
$>5\times10^5$	20	396	-1.10 [-1.43, -0.76]		
$(1\text{-}5)\times10^5$	21	410	-1.88 [-2.35, -1.40]	0.03	2.66%
$<1\times10^5$	11	239	-1.53 [-2.22, -0.84]		
Type of manipulation of stem cells prior to implantation					
Gene-modification	17	354	-1.46 [-1.88, -1.03]	0.91	N/A

None	35	691	-1.49 [-1.84, -1.13]		
Quality Scores					
11	3	81	-1.05 [-1.52, -0.58]		
12	9	179	-0.75 [-1.07, -0.42]		
13	15	259	-1.52 [-1.94, -1.09]		
14	17	331	-1.98 [-2.55, -1.41]	<0.0001	N/A
15	5	95	-3.01 [-4.88, -1.13]		
16	2	60	-0.39 [-0.95, 0.17]		
18	1	40	-0.70 [-1.34, -0.06]		
Route of delivery					
Stereotaxic	42	820	-1.54 [-1.87, -1.21]	0.30	N/A
Systemic	10	225	-1.25 [-1.68, -0.83]		

Note: *P* value for test for subgroup differences. Adjusted R^2 for study characteristics accounting for heterogeneity.

3.2 Memory capability: Stratified analysis of stem cell-treated *vs*. control

	Number of comparisons	Number of Participants	Std. Mean difference (IV, random,95%CI)	*P*	Adjusted R^2
Pooled estimate	73	1495	1.27 [1.04, 1.49]		
AD model					
Transgenic	40	906	0.99 [0.73, 1.25]	0.01	28.35%
Aβ- infused	21	381	1.77 [1.33, 2.20]		
F-F transcation	2	36	4.14 [1.21, 7.06]		
Natural senile	2	40	1.07 [0.39, 1.74]		
Chem-infused	8	132	1.04 [0.38, 1.69]		
Recipient species					
TG	38	826	0.96 [0.69, 1.24]	0.007	16.96%
Rat	23	395	1.82 [1.29, 2.35]		
Mouse	12	274	1.43 [1.11, 1.74]		
Recipient sex					
Both	2	38	0.30 [-0.35, 0.94]	0.003	15.85%
Male	56	1157	1.44 [1.16, 1.72]		

Female	2	270	0.93 [0.66, 1.20]		
Unclear	13	30	0.70 [-0.05, 1.45]		
Donor species					
Mouse	26	546	0.88 [0.54, 1.22]		
Human	23	564	1.21 [0.93, 1.48]	0.004	26.22%
Rat	15	254	2.50 [1.70, 3.30]		
Cell line	9	131	1.16 [0.57, 1.75]		
Type of stem cells					
MSCs	41	880	1.13 [0.89, 1.37]		
NSCs	23	481	1.34 [0.92, 1.77]	0.38	N/A
NPCs	9	134	2.24 [0.96, 3.52]		
Number of cells injected					
>5×10^5	34	695	0.91 [0.70, 1.11]		
(1-5) ×10^5	25	494	1.81 [1.37, 2.26]	0.008	14.63%
<1×10^5	14	306	1.49 [0.79, 2.18]		
Quality Scores					
11	3	74	1.22 [0.53, 1.92]		
12	12	238	0.70 [0.43, 0.96]	0.004	N/A
13	26	481	1.31 [0.97, 1.65]		
14	18	348	1.98 [1.37, 2.59]		

15	8	154	1.16 [0.23, 2.08]		
16	4	120	0.96 [0.00, 1.92]		
18	2	80	1.46 [0.76, 2.15]		
Type of manipulation of stem cells prior to implantation					
Gene-modification	26	586	1.16 [0.88, 1.44]	0.36	N/A
None	47	909	1.36 [1.04, 1.68]		
Route of delivery					
Stereotaxic	53	1041	1.31 [1.01, 1.60]	0.80	N/A
Systemic	20	454	1.26 [1.01, 1.50]		

Note: *P* value for test for subgroup differences. Adjusted R^2 for study characteristics accounting for heterogeneity.
